# Incremental induction of NMDAR-STP and NMDAR-LTP in the CA1 area of ventral hippocampal slices relies on graded activation of discrete NMDA receptors

**DOI:** 10.1098/rstb.2023.0239

**Published:** 2024-07-29

**Authors:** Rachael Ingram, Rasa Volianskis, John Georgiou, David E. Jane, Adina T. Michael-Titus, Graham L. Collingridge, Arturas Volianskis

**Affiliations:** ^1^ Centre for Neuroscience, Surgery and Trauma, Blizard Institute, Barts and The London School of Medicine and Dentistry, Queen Mary University of London, London, UK; ^2^ Lunenfeld-Tanenbaum Research Institute, Mount Sinai Hospital, Sinai Health System, Toronto, Ontario, Canada; ^3^ Department of Physiology, University of Toronto, Toronto, Ontario, Canada; ^4^ TANZ Centre for Research in Neurodegenerative Diseases, University of Toronto, Toronto, Ontario, Canada; ^5^ Hello Bio Limited, Cabot Park, Avonmouth, Bristol, UK; ^6^ School of Biosciences, Cardiff University, Museum Avenue, Cardiff, UK

**Keywords:** short-term potentiation, long-term potentiation, NMDA receptor, ventral hippocampus, dorsal hippocampus, synaptic plasticity

## Abstract

*N*-methyl-d-aspartate receptor (NMDAR)-dependent short- and long-term types of potentiation (STP and LTP, respectively) are frequently studied in the CA1 area of dorsal hippocampal slices (DHS). Far less is known about the NMDAR dependence of STP and LTP in ventral hippocampal slices (VHS), where both types of potentiation are smaller in magnitude than in the DHS. Here, we first briefly review our knowledge about the NMDAR dependence of STP and LTP and some other forms of synaptic plasticity. We then show in new experiments that the decay of NMDAR-STP in VHS, similar to dorsal hippocampal NMDAR-STP, is not time- but activity-dependent. We also demonstrate that the induction of submaximal levels of NMDAR-STP and NMDAR-LTP in VHS differs from the induction of saturated levels of plasticity in terms of their sensitivity to subunit-preferring NMDAR antagonists. These data suggest that activation of distinct NMDAR subtypes in a population of neurons results in an incremental increase in the induction of different phases of potentiation with changing sensitivity to pharmacological agents. Differences in pharmacological sensitivity, which arise due to differences in the levels of agonist-evoked biological response, might explain the disparity of the results concerning NMDAR subunit involvement in the induction of NMDAR-dependent plasticity.

This article is part of a discussion meeting issue ‘Long-term potentiation: 50 years on’.

## 1. Introduction

### (a) Different *N*-methyl-d-aspartate receptor (NMDAR)-dependent forms of synaptic plasticity: short-term potentiation (STP), long-term potentiation (LTP) and long-term depression (LTD)

Activity-dependent potentiation and depression of synaptic transmission are thought to underlie the encoding of memories in the brain, and a variety of distinct types of synaptic plasticity have been described [1]. Different forms of synaptic plasticity have been classified in a number of ways: (i) based on their duration [2–4], (ii) neurotransmitter systems and ion channels that contribute to their induction [5–7], and (iii) second messenger systems that contribute to their expression and maintenance [3,4,6–8].

NMDAR-dependent forms of synaptic plasticity, which include short-term potentiation (STP), long-term potentiation (LTP) and long-term depression (LTD), are some of the most frequently studied types of synaptic plasticity [1,5,9]. STP, LTP and LTD rely on the activation of NMDARs during their induction and have relatively long-lasting effects on the strength of synaptic transmission (>30 min to years), making them attractive physiological candidates for the storage of memories [2,5,10]. During the 50 years since the discovery of LTP [11,12], LTP and LTD have attracted a lot of research attention, establishing themselves as putative correlates of long-term memory [10,13]. NMDAR-dependent STP (NMDAR-STP) has attracted less experimental interest than LTP and LTD, with some significant confusion in the literature.

### (b) The muddle about STP

A lot of the mix-up with regard to NMDAR-STP comes from the fact that some other forms of synaptic plasticity, such as paired-pulse facilitation (PPF), frequency facilitation (FF) and post-tetanic potentiation (PTP), are known under the umbrella term ‘short-term plasticity’ [4], with the unfortunate consequence of sharing the acronym ‘STP’ with NMDAR-STP. PPF, FF and PTP are shorter-lasting (milliseconds, seconds or a few minutes) than NMDAR-STP (minutes to hours) and, importantly, do not require NMDAR involvement for their induction [4,14–16]. The phenomena of short-term plasticity may be involved in reverberating (or persistent) activity and working memory formation [17–22], somewhat similarly to NMDAR-STP, which is also thought to be involved in shorter-lasting memories when compared to LTP [23–25].

Adding to the confusion about the acronym is the fact that the differentiation of NMDAR-dependent STP (short-term potentiation) from NMDAR-independent STP (short-term plasticity) is a fairly recent development. Indeed, prior to the 1983 discovery of the role of NMDARs in LTP [26], most of the exponentially decaying potentiation phenomena, which can be observed at both the neuromuscular junction and at central synapses, were known as PTP or facilitation [4,20,27,28], albeit with some exceptions [29]. The separation of NMDAR-STP from PTP became more widely accepted after the 1993 review by Bliss and Collingridge, which divided plasticity into NMDAR-dependent and NMDAR-independent types [1]. It was much later that it was shown that NMDAR-STP and PTP are fundamentally different: (i) PTP in hippocampal synapses decays within ~2 min, while NMDAR-STP lasts ~20 min or more in most experiments [23,30], (ii) PTP is independent of calcium/calmodulin-dependent protein kinase II (CAMKII), while NMDAR-STP has been shown to depend on CAMKII [30,31], and (iii) the decay of PTP is time-dependent [23,32], while NMDAR-STP decays in response to pre-synaptic activation—it is actively de-potentiated by stimulation [23,33]. Indeed, NMDAR-STP appears to be more similar to NMDAR-LTP than to the NMDAR-independent types of synaptic plasticity, and therefore, due to its transiently decaying nature, NMDAR-STP has been termed transient-LTP [23]. However, the field did not agree with this change of the name, and the muddle with the nomenclature continues unresolved. To avoid any further ambiguity, throughout this article, we will refer to short-term potentiation as NMDAR-STP, in contrast to the NMDAR-independent PTP and other NMDAR-independent forms of short-term plasticity. We will refer to NMDAR-dependent LTP as LTP.

### (c) NMDAR-STP and LTP in the CA1 area of the Schaffer collaterals in the hippocampus

**Figure 1.**
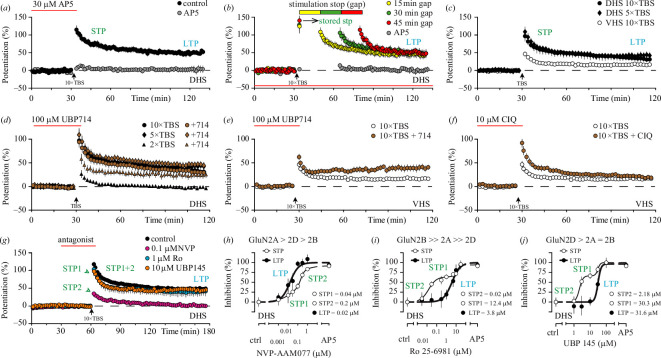
NMDAR dependence of NMDAR-STP and LTP in the hippocampus (previously published [5,34,35]). (*a*) Mean time course of potentiation (black circles ± standard error of the mean (s.e.m.)) induced by 10× TBS in DHS. NMDAR-dependent STP declined to a steady level of LTP in about 1 h (data from [5]). Application of AP5 (red line) inhibited the induction of both NMDAR-STP and LTP (grey circles; data from [35]). (*b*) Decay of NMDAR-STP is not time-dependent, and its levels can be maintained during periods without stimulation (yellow, 15 min; green, 30 min; red, 45 min; data from [5]). Grey circles show an experiment in which the induction of NMDAR-STP and LTP was inhibited by AP5 (data from [35]).(*c*) 10× TBS induces smaller NMDAR-STP and LTP in VHS (white circles) when compared to both 5× and 10× TBS in DHS (data from [34]). (*d*) GluN2A/2B potentiator UBP714 (brown symbols) facilitates the induction of LTP in 2× TBS experiments but not in 5× or 10× TBS experiments in DHS (data from [34]). (*e*) UBP714 (brown circles) shortens the decay of NMDAR-STP and facilitates induction of LTP in 10× TBS experiments in VHS (data from [34]). (*f*) GluN2C/D potentiator CIQ (brown circles) facilitates the induction of larger NMDAR-STP and prolongs its decay in VHS. CIQ does not enhance LTP (data from [34]). (*g*) GluN2A-preferring antagonist NVP-AAM077 (NVP, pink circles) blocks LTP and inhibits fast STP1 while preserving STP2. GluN2B antagonist Ro 25-6981 (Ro, light blue circles) and GluN2C/2D-prefering antagonist UBP145 (orange circles) inhibit slow STP2 and do not affect STP1 or LTP. Control is shown in black (all data from [35]). (h,i and *j*) Full concentration response curves for inhibition of STP1, STP2 and LTP by NVP, Ro and UBP145 in DHS. The rank-order potency of the antagonists for the different GluN2 subunits was determined in HEK293 cells (all data replotted from [35]).

NMDAR-STP and LTP are frequently co-induced at hippocampal synapses (figure 1*a* [23]), but they can also be induced independently of each other [29,36–38]. In contrast to LTP, which shifts the level of synaptic transmission towards potentiation in a static fashion [39,40], NMDAR-STP is dynamic [24]. Thus, NMDAR-STP modulates the synaptic frequency response [24], similar to presynaptic forms of LTP [41–43], while post-synaptic LTP preserves the fidelity of the response and amplifies neural transmission [39,40]. NMDAR-STP increases during brief high-frequency bursts of activity and declines exponentially in response to infrequent synaptic activation, either to baseline or to a sustained level of LTP (figure 1*a* [23,27,29,44]). The rate of NMDAR-STP decay is directly related to the number of de-potentiating stimuli, such that NMDAR-STP decays faster in experiments with more frequent afferent stimulation than with slower stimulation [23,27,29]. Thus, during periods of synaptic inactivity following the induction, the level of NMDAR-STP is ‘stored’ in synapses, providing temporal stability in synaptic strength (figure 1*b*; see also [33]). Storage of NMDAR-STP during periods of synaptic inactivity has been demonstrated for up to 6 h in hippocampal slices [23], making it an attractive mechanism for storage of such memories that can neither be sustained by a reverberatory trace nor by a semi-permanent structural alteration [21]. Differently from LTP, which saturates after ~2 s of theta-burst stimulation (TBS) [45], NMDAR-STP can be repeatedly re-induced and de-potentiated under the baseline conditions [29], and after the saturation of LTP [28,36].

NMDAR-STP, LTP and LTD are most commonly studied in dorsal hippocampal slices (DHS) [23,26,37,46–48], although they can be induced in many other brain areas [27,28,49–52]. Interestingly, much smaller LTP and larger LTD have been reported in ventral hippocampal slices (VHS) when compared to dorsal [53–57], and most of the above-mentioned literature on LTP suggests that NMDAR-STP is smaller in the ventral hippocampus than in the dorsal. The expression of NMDAR-independent forms of plasticity also differs significantly when compared across the dorsoventral hippocampal axis [58–61]. The reasons for such intrahippocampal differences are not completely clear, and a variety of explanations have been suggested.

### (d) Effects of NMDAR potentiators on NMDAR-STP and LTP in dorsal and ventral hippocampal slices

We have recently shown that NMDAR-STP, similar to LTP, is indeed smaller in VHS than in DHS (figure 1*c* [34]) and reported some interesting pharmacological observations with respect to the effects of NMDAR subunit-preferring potentiators on the induction of both NMDAR-STP and LTP [34]. Notably, in experiments in DHS, in which two 4-pulse bursts (100 Hz) were delivered at theta frequency (2× TBS [45,62]), the GluN2A/2B positive allosteric modulator (PAM; table 1) UBP714 facilitated the induction of LTP but not NMDAR-STP, which did not change in its amplitude or decay time constant (figure 1*d*). In these experiments, submaximal levels of NMDAR-STP and LTP were observed. However, in experiments in which either 5× or 10× TBS induced maximal levels of potentiation, the UBP714 effect on LTP was no longer observed (figure 1*d*). These results demonstrate that subsaturated levels of LTP can be potentiated by enhancing NMDAR function. In VHS, where the level of LTP was lower than in DHS (figure 1*c*), UBP714 facilitated the 10× TBS-induced LTP and decreased the duration of NMDAR-STP, which would suggest sub-saturated LTP under the control conditions in the ventral slices (figure 1*e*). Both the magnitude of NMDAR-STP and its duration could be increased in VHS by the GluN2C/D potentiator CIQ (table 1), which had no effect on the induction of LTP (figure 1*f*). The ability of UBP714 to speed up the decay of NMDAR-STP and the ability of CIQ to prolong the duration of NMDAR-STP are particularly noteworthy, as it has been reported previously that the duration of STP varies significantly between different limbic areas, with two distinct types of STP—a fast and a slow being readily discernible [27].

**Table 1.** Pharmacological characterization of NMDAR subunit-preferring compounds.

subunits / compounds	GluN2A^(recombinant)^	GluN2B^(recombinant)^	GluN2C^(recombinant)^	GluN2D^(recombinant)^	NMDARs^(neurons)^
D-AP5^CA^	0.28^a^/1.06^b^	0.46^a^/2.68^b^	1.64^a^	3.71^a^/44.9^b^	0.6^a^
CIQ^PAM^	>10^c^	>10^c^	2.7^c^	2.8^c^	130^c^
NVP-AAM077^CA^	0.0054^a^/0.048^b^	0.067^a^/0.6^b^	0.012^a^	0.037^a^/0.1^b^	0.033^b^
Ro 25-6981^NAM^	52–250^b^	0.009–0.057^b^	unknown	no inhibition	1.22^b^
UBP145^CA^	11.5^a^/16^b^	8.0^a^/13^a^	2.8^a^	1.19^a^/1.3^b^	11.5^b^
UBP714^PAM^	17^d^	14^d^	unknown	4.4^d^	17^d^

*Notes:* NMDAR compounds that are discussed in the current study. All data are given in µM. D-AP5 data from [35,63]. CIQ data from [64]. Please note that potentiation of native NMDARs is for the subthalamic neurons, and no potentiation was seen for the CA1 pyramidal cells. NVP-AAM077 data from [35,65]. Please note that in [65], NVP is referred to as PEAQX. Ro 25-6981 data from [35,66]. UBP145 data from [35,67]. UBP714 data from [68].

^a^ *K*_*i*_ values.

^b^ IC_50_ values.

^c^ EC_50_ values.

^d^ Percentage of potentiation above control levels.

CA, competitive antagonists; NAM, negative allosteric modulator; PAM, positive allosteric modulator.

The differing effects of UBP714 and CIQ on NMDAR-STP and LTP in VHS also suggest that induction of these two forms of potentiation depends on discrete NMDAR subtypes, which is consistent with previous observations using different subunit-preferring NMDAR antagonists in DHS [35,69]. Indeed, a fast and a slow NMDAR-STP, which we termed STP1 and STP2, respectively, are sensitive to different subunit-preferring NMDAR antagonists in DHS (figure 1*g* [35]). The fast STP1 and LTP are particularly sensitive to GluN2A preferring antagonists NVP-AAM077 (NVP; figure 1*g,h*) and AP5 [35,37]. The slow STP2 is particularly sensitive to the GluN2B antagonist Ro 25-6981 (Ro; figure 1*g,i*) and the GluN2C/D antagonist UBP145 (figure 1*g,j*). These antagonists have been characterized in detail [35,63–68], both in recombinant receptor systems and against native NMDARs in DHS (table 1). The effects of these antagonists on NMDAR-STP or LTP in VHS have not been studied previously, and it is still unknown whether the levels of NMDAR-STP, which are induced in VHS, can be stored during pauses in the stimulation.

In the present study, we have characterized NMDAR-STP in VHS. First, we have examined the effects of altering the number of TBS on the induction of NMDAR-STP and LTP and the effects of a pause in stimulation on the maintenance of potentiation. Second, we have examined the sensitivity of ventral hippocampal NMDAR-STP and LTP to the same subtype-selective NMDAR antagonists (NVP, Ro and UBP145) as in our previous studies in the DHS [35,69]. Third, we have explored how the sensitivity to these antagonists is influenced by the number of TBS delivered. We describe here that in VHS, NMDAR-STP and LTP differ in their sensitivity to NMDAR antagonists. We also show that the duration of NMDAR-STP can be reliably modulated by the number of bursts delivered during TBS and that the fast and slow types of NMDAR-STP (STP1 and STP2), induced by the specific paradigms, demonstrate differential sensitivity to some of the NMDAR antagonists. A graded induction of LTP, which increased in its sensitivity to NMDAR antagonists with stronger TBS, was also observed in VHS. These observations suggest that discrete NMDA receptors, activated by specific induction stimuli in a population of synapses, are responsible for the additive induction of specific types of potentiation.

## 2. Material and methods

### (a) Slice preparation, electrophysiological recordings and chemicals

Experiments were performed after institutional approval, according to the UK Scientific Procedures Act, 1986 and European Union guidelines for animal care. Animals (male Wistar rats, 200–220 g; Charles River Laboratories, UK) were sacrificed by cervical dislocation after isoflurane anaesthesia (Schedule 1). Transverse slices (400 μm) were cut from either the dorsal or the ventral pole of the hippocampus using a McIllwain tissue chopper, according to the procedures that were described previously in detail [23,35]. A total of 87 rats were used, producing 141 DHS and VHS recordings, as reported in this paper.

Slices were pre-incubated at room temperature in artificial cerebrospinal fluid (ACSF) containing (in mM) NaCl (130), D-Glucose (10), NaHCO_3_ (26), KCl (3.5), NaH_2_PO_4_ (1.2), MgSO_4_ (7H_2_O) (2) and CaCl_2_ (2), for at least 2 h prior to the start of the experiments. During the experiments, the slices were perfused at a rate of 2.5 ml/min and maintained submerged in ACSF (32°C). ACSF was saturated with 95% O_2_–5% CO_2_, in all conditions.

The Schaffer collaterals were stimulated using a platinum/iridium concentric bipolar electrode (CBAPB50; FHC, Inc., USA) placed on the border between CA3 and CA2, in the stratum radiatum. Extracellular field excitatory post-synaptic potentials (fEPSPs) were recorded from the CA1 area of the stratum radiatum, using ACSF-filled borosilicate glass electrodes (1.5–3.5 MΩ). fEPSPs were amplified (MultiClamp 700A; Axon Instruments), filtered at 3 kHz and digitized at 40 kHz (National Instruments, PCIe-6321). The stimulation current (A385; WPI) was set to three times the threshold current to elicit fEPSPs. WinLTP software (www.winltp.com) was used to control the timing of the experiments and to visualize and record fEPSPs, which were stored on a PC [70].

Test stimulation was given once every 15 s (0.067 Hz), both before and after the induction of potentiation, and fEPSPs were recorded as the mean of four responses over a period of 1 min. Stability of baseline responses was monitored for at least 45 min prior to the induction of potentiation. Potentiation was induced by TBS: four pulses delivered at 100 Hz, repeated either 10, 30 or 100 times at a 5 Hz frequency (10× TBS, 30× TBS or 100× TBS). In all experiments, stimulation was interrupted for 3 min post-TBS to avoid PTP affecting the measurements of NMDAR-dependent plasticity [23]. In experiments, in which NMDAR antagonists were used, compounds were bath applied for 30 min, after a 15 min recording of baseline potentials. Compounds were washed out following the TBS. Experiments were performed in an interleaved manner, randomizing the application of different compounds and induction protocols.

NVP-AAM077 (NVP) and UBP145 were synthesized at the University of Bristol. Ro 25-6981 (Ro) and AP5 were purchased from Abcam (Cambridge, UK). All compounds were prepared as stock solutions, stored at −20°C and diluted into ACSF during the experiments. Detailed characterization of these compounds has been published previously [35,65,66,71].

### (b) Analysis of electrophysiological recordings and statistical analysis

The fEPSPs from individual experiments were quantified off-line by measuring the rate of rise (mV/ms) of their early initial slope (0.25 ms duration), after the termination of the fibre volley, corresponding to the steepest part of the fEPSP (confirmed by differentiation of the responses; Platin Calculator, Morten S. Jensen, Aarhus University, Denmark). The data were normalized to the baseline period, which was set at 100%, reflecting relative changes in the strength of synaptic transmission.

NMDAR-STP is sometimes [23,35], but not always [27,35,44], induced as a uniformly decaying single-exponential phenomenon. Due to the noise levels of individual experiments and the redundancy of mathematical models, double-exponential functions cannot be used to reliably quantify the results, while single-exponential functions discriminate reliably between fast- and slow-decaying NMDAR-STPs. Therefore, individual normalized experiments were curve fitted using a single-exponential decay function (Potentiation amplitude = LTP + STP e^*−t*/*τ*^), as described previously in [23], estimating the amplitudes of NMDAR-STP (%) and LTP (%), as well as the decay time constant of NMDAR-STP (*τ*, min). Statistical analyses of these parameters are reported in Results, comparing the effects of different induction protocols on synaptic plasticity.

To estimate the inhibitory effects of the NMDAR antagonists on the induction of plasticity, NMDAR-STP was additionally quantified as the area under the decaying curve in individual experiments (NMDAR-STP_Area_ = NMDAR-STP amplitude × *τ*). The percentage inhibition of NMDAR-STP_Area_ could then be calculated, relative to the mean of the control without the application of antagonists, as described previously in DHS [35]. Similarly, the percentage inhibition of LTP_Level_ was calculated in individual experiments relative to the mean LTP amplitude without the application of antagonists. The inhibition of NMDAR-STP_Area_ and LTP_Level_ by the antagonists is reported and compared in Results.

Time courses of potentiation are presented in Results as mean values of potentiation (%) ± standard error of the mean (s.e.m.), plotted over time (h or min). For presentation, the individual data points are averaged over 2 min, with baseline levels subtracted. Results of all parameters and calculations are reported as mean values ± s.e.m. Unpaired two-tailed *t*-tests and ANOVAs with Tukey’s or Dunnett’s multiple comparison tests were used for the between-groups statistics (GraphPad Prism). Additionally, for more detailed presentation of the different potentiation components, some of the mean experimental datasets were fitted with either single- or double-exponential functions, and these results are visualized in figure 5 . *F*-test was used to determine whether single- or double-exponential fit was most appropriate for the data (GraphPad Prism).

## 3. Results

### (a) Incremental induction of NMDAR-STP and LTP in ventral hippocampal slices

**Figure 2.**
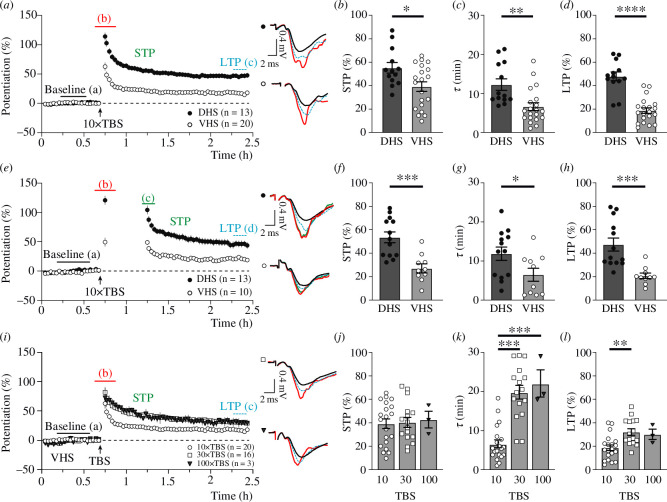
The magnitude of hippocampal NMDAR-STP and LTP is dependent on slice preparation and the number of theta bursts delivered. (*a*) Pooled data showing the time courses of potentiation of fEPSPs (mean ± s.e.m.) for DHS (black circles) and VHS (white circles). The coloured letters depict the timing of the fEPSPs, shown to the right. (*b*) The amplitude of NMDAR-STP was significantly greater in DHS compared to VHS (55.3 ± 4.5% versus 39.2 ± 4.1%; *p* = 0.016). (*c*) The decay time constant of NMDAR-STP was greater in DHS (12.3 ± 1.5 min) than in VHS (6.6 ± 1.0 min; *p* = 0.0025). (*d*) The LTP was larger in the DHS compared to the VHS (47.9 ± 3.7% versus 18.8 ± 2.3%; *p* < 0.0001). (*e*) NMDAR-STP is stored when test stimulation is paused for 30 min in both DHS (black circles) and VHS (white circles). (*f*) The amplitude of stored NMDAR-STP is greater in DHS compared to VHS (53.6 ± 4.6% versus 27.1 ± 3.9%; *p* = 0.00037). (*g*) NMDAR-STP decayed at a slower rate in DHS (11.9 ± 1.7 min) than in VHS (6.5 ± 1.7 min; *p* = 0.039). (*h*) LTP was greater in DHS compared to VHS (47.4 ± 5.5% versus 20.7 ± 2.4%; *p* = 0.00064). (*i*) Time course of NMDAR-STP and LTP induced by 10× TBS (white circles; results from panel *a* are reproduced in panel i), 30× TBS (white squares) and 100× TBS (grey triangles) in VHS. (*j*) The amplitude of NMDAR-STP was similar for all three induction paradigms: 39.2 ± 4.1% (10× TBS), 40.3 ± 4.3% (30× TBS) and 42.7 ± 7.1% (100× TBS; *p* = 0.94, ANOVA). (*k*) The rate of NMDAR-STP decay was significantly faster in the 10× TBS group (6.6 ± 1.0 min) compared to the 30× TBS experiments (19.9 ± 1.7 min; *p* < 0.0001, Tukey’s multiple comparisons test) and the 100× TBS group (22.1 ± 3.5 min; *p* = 0.0003, Tukey’s multiple comparisons test). (*l*) LTP was greater in the 30× TBS (32.2 ± 2.8%) group compared to 10× TBS (18.8 ± 2.3%, *p* = 0.0016, Tukey’s multiple comparisons test), while 100× TBS did not induce any more LTP (30.2 ± 4.4%, *p* = 0.95, Tukey’s multiple comparisons test).

Ten 4-pulse 100 Hz bursts, delivered at a 5 Hz frequency (10× TBS), are thought to be optimal for inducing maximal levels of potentiation in DHS (figure 1*d* [23,45,62,72]). In the current DHS experiments, 10× TBS induced NMDAR-STP (55.3 ± 4.5%) that declined with a *τ* value of 12.3 ± 1.5 min to a 47.9 ± 3.7% level of LTP (figure 2, black circles). The application of 10× TBS in the VHS resulted in a smaller NMDAR-STP (39.2 ± 4.1%) that declined faster (*τ* = 6.6 ± 1.0 min) to a lower level of LTP (18.8 ± 2.3%) than in the DHS (figure 2*a–d*, open and black circles, respectively). In both DHS and VHS, the decline of NMDAR-STP was use-dependent but not time-dependent, in that a 30 min delay in stimulation suspended the decline of NMDAR-STP (figure 2*e*, black circles versus white circles). This suggests that the process of NMDAR-STP storage might be similar in DHS and VHS. The differences in the magnitudes of NMDAR-STP and LTP between DHS and VHS were not affected by the introduction of a delay in baseline stimulation (figure 2*f–h*).

We next tested whether greater amounts of potentiation could be induced in VHS by increasing the number of theta bursts during the induction (figure *2i,l*). The amplitude of the 30× TBS-induced NMDAR-STP (40.3 ± 4.3%; figure 2*i,j*, white squares) was similar to the 10× TBS-induced NMDAR-STP (39.2 ± 4.1%), but it declined with a much slower decay time constant (19.9 ± 1.7 min versus 6.6 ± 1.0 min, 30× and 10× TBS, respectively; figure 2*k*). Significantly larger LTP was induced by 30× TBS than by 10× TBS (32.2 ± 2.8% versus 18.8 ± 2.3%, 30× and 10× TBS, respectively; figure 2*l*). Such dependence of both *τ* and the magnitude of LTP on the number of stimuli during trains of stimulation has been previously reported in DHS [23]. We have therefore tested whether a further increase in the number of theta bursts would result in greater potentiation in VHS. However, the magnitudes of NMDAR-STP (42.7 ± 7.1%; figure 2*j*) and LTP (30.2 ± 4.4%; figure 2*l*) and the decay time of NMDAR-STP (22.1 ± 3.5 min; figure 2*k*), which were recorded in response to 100× TBS (figure 2*i*; grey triangles), were very similar to those in 30× TBS (figure 2*i–l*).

In summary, on the basis of the above-mentioned experiments, we conclude that subsaturated levels of NMDAR-STP and LTP are induced in VHS by a 10× TBS protocol. A dramatic slowing down in the decay of NMDAR-STP and an increase in the levels of LTP are observed with the stronger stimulation protocols. This suggests that incremental induction of NMDAR-STP and LTP leads to saturation of the potentiation processes in the VHS.

### (b) Sensitivity of ventral NMDAR-STP and LTP to GluN2 subunit-preferring NMDAR antagonists

Differential sensitivity of NMDAR-STP and LTP to GluN2 subunit-preferring NMDAR antagonists NVP, Ro and UBP145 (table 1) has been observed in DHS (figure 1*g–j* [35]). Here, fast-decaying NMDAR-STP and LTP were particularly sensitive to low concentrations of NVP (10–100 nM), which show greatest selectivity to GluN2A subunits. In contrast, low concentrations of Ro (GluN2B-selective) and UBP145 (GluN2D-preferring) inhibited the induction of the slow-decaying NMDAR-STP and did not affect the induction of fast-decaying NMDAR-STP or LTP (figure 1*g–j*).

NVP, Ro and UBP145 have not been tested on the induction of potentiation in VHS. Based on the previous results from DHS and on the observation that 30× TBS prolongs the decay of NMDAR-STP in VHS when compared to 10× TBS (figure *2i,l*), we can predict a greater sensitivity of the 10× TBS-induced fast-decaying NMDAR-STP to NVP than when tested with 30× TBS. Furthermore, we also predict a greater sensitivity of the 30× TBS-induced slow-decaying NMDAR-STP to Ro and UBP145 than with 10× TBS.

**Figure 3.**
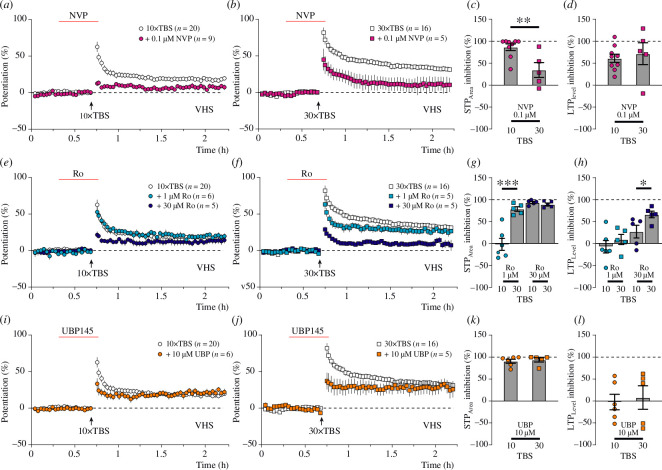
Discrete sensitivity of NMDAR-STP and LTP to different NMDAR antagonists in VHS. (*a*) Time course of potentiation induced by 10× TBS in the presence of 0.1 μM GluN2A-preferring antagonist NVP (pink circles) and the control experiments (white circles). (*b*) Time course of NMDAR-STP and LTP induced by 30× TBS in the presence of 0.1 μM NVP (pink squares) and in controls (white squares). (*c*) NMDAR-STP_Area_ (STP_Area_) induced by 10× TBS was inhibited to a greater extent by 0.1 μM NVP compared to the 30× TBS group with 86.5 ± 7.3% and 34.4 ± 16.9% inhibition, respectively (*p* = 0.0063). (*d*) NVP inhibited LTP_Level_ induced by 10× TBS (61.2 ± 9.4%) and 30× TBS (71.6 ± 24.9%) to a similar extent (*p* = 0.64). (*e*) Potentiation induced by 10× TBS in the presence of 1 μM (light blue circles) and 30 μM (dark blue circles) Ro. (*f*) NMDAR-STP and LTP induced by 30× TBS with the application of 1 μM (light blue squares) and 30 μM (dark blue squares) GluN2B-preferring antagonists, Ro. (*g*) NMDAR-STP_Area_ induced by 10× TBS was not sensitive to 1 μM Ro (−1.9 ± 13.6% inhibition), whereas NMDAR-STP_Area_ induced by 30× TBS was highly sensitive to 1 μM Ro (78.7 ± 5.2% inhibition, *p* = 0.00064). Ro at 30 μM inhibited NMDAR-STP_Area_ induced by both 10× and 30× TBS similarly (93.5 ± 2.4% and 89.3 ± 3.1%, *p* = 0.31). (*h*) 1 μM Ro did not significantly inhibit LTP_Level_, and its effects were similar when compared between 10× and 30× TBS (−7.4 ± 14.2% and 9.1 ± 11.6%, *p* = 0.40). LTP_Level_ induced by 30× TBS was more sensitive to the presence of 30 μM Ro (66.3 ± 7.5%) than 10× TBS-induced LTP (27.1 ± 14.5%, *p* = 0.043). (*i*) NMDAR-STP and LTP induced by 10× TBS in the presence of 10 μM GluN2D-preferring antagonist, UBP145 (orange circles). (*j*) Potentiation induced by 30× TBS in the presence of 10 μM UBP145 (orange squares). (*k*) 10 μM of UBP145 inhibited 10× and 30× TBS-induced NMDAR-STP_Area_ to a similar extent (90.7 ± 4.5% and 93.7 ± 4.9% inhibition, respectively, *p* = 0.66). (*i*) UBP145 did not inhibit the induction of LTP_Level_, and its effects were similar when compared between 10× TBS (−2.5 ± 17.7% inhibition) and 30× TBS groups (8.0 ± 26.8% inhibition, *p* = 0.75).

Much smaller NMDAR-STP (8.6 ± 2.2%, *τ* = 2.6 ± 1.1 min) and LTP (7.3 ± 1.8%) were induced by 10× TBS (figure 3, pink circles) in experiments in which 0.1 μM NVP was applied for 30 min prior to tetanization, when compared to the controls (figure 3*a*, black circles; NMDAR-STP = 39.2 ± 4.1%, *τ* = 6.6 ± 1.0 min; LTP = 18.8 ± 2.3%). In contrast, a substantial slow-decaying NMDAR-STP (31.2 ± 8.7%, *τ =* 17.8 ± 4.1 min) was induced by 30× TBS (figure 3*b*, pink squares) in the presence of 0.1 μM NVP, while LTP (9.1 ± 8.0%) was greatly inhibited when compared with the experiments without the antagonist (figure 3*b*, black squares; NMDAR-STP = 40.3 ± 4.3%, *τ* = 19.9 ± 1.7 min; LTP = 32.2 ± 2.8%). To assess these results quantitatively, we calculated the percentage inhibition of NMDR-STP_Area_ (figure 3*c*) and the percentage inhibition LTP_Level_ (figure 3*d*) and compared their inhibition between the 10× and the 30× TBS groups. Thus, 0.1 μM NVP-inhibited NMDAR-STP_Area_ by 86.5 ± 7.3% in the 10× TBS group compared to only 34.4 ± 16.9% in the 30× TBS experiments (figure 3*c*). The inhibition of LTP_Level_ by NVP in the 10× TBS group (61.2 ± 9.4%) was similar to that in 30× TBS (71.6 ± 24.9%; figure 3*d*). These results support the prediction that the sensitivity of NMDAR-STP to the GluN2A-preferring concentration of NVP decreases when the decay time constant of NMDAR-STP increases.

Application of 1 μM Ro (figure 3*e*, light blue circles) in 10× TBS experiments had no effect on the induction of NMDAR-STP (32.0 ± 5.0%*, τ* = 10.8 ± 2.9 min) or LTP (20.2 ± 2.7%), when compared to the control experiments without the application of the antagonist (figure 3*e*, white circles; NMDAR-STP = 39.2 ± 4.1%, *τ* = 6.6 ± 1.0 min; LTP = 18.8 ± 2.3%). However, in the presence of a higher concentration of Ro (30 μM, dark blue circles), both NMDAR-STP (9.3 ± 1.9%, *τ* = 2.2 ± 0.9 min) and LTP (13.7 ± 2.7%) were smaller than in the control (figure 3*e*, white circles). Effects of 1 μM Ro on the induction of NMDAR-STP became apparent in 30× TBS experiments (figure 3*f*, white versus light blue squares) in which NMDAR-STP (32.0 ± 5.3% versus 40.3 ± 4.3%) declined substantially faster (5.2 ± 1.1 min versus 19.9 ± 1.7 min) than in the controls, while LTP was largely unaffected (29.2 ± 3.7% versus 32.2 ± 2.8%, 1 μM Ro and controls, respectively). Both NMDAR-STP (9.3 ± 1.9%, *τ* = 2.2 ± 0.9 min) and LTP (13.7 ± 2.7%) were inhibited by 30 μM Ro in 30× TBS experiments (figure 3*f*, dark blue versus white squares). Analyses of percentage inhibition of NMDAR-STP_Area_ (figure 3*g*) showed that 1 μM Ro inhibited NMDAR-STP more potently in experiments with 30 than with 10× TBS. Similarly, 30 μM Ro inhibited 30× TBS-induced LTP more potently than 10× TBS-induced LTP (figure 3*h*). These results support the prediction that slow-decaying NMDAR-STP is more sensitive to the GluN2B antagonist Ro than fast-decaying NMDAR-STP. These experiments also show that the sensitivity of LTP to Ro increases as the magnitude of LTP gets larger. However, although the sensitivity of both NMDAR-STP and LTP to the GluN2B antagonist increases as the number of theta bursts increases, a much higher concentration of Ro is needed to inhibit LTP than NMDAR-STP.

In contrast to NVP and Ro, application of 10 μM of UBP145 did not produce any differential effects on the induction of NMDAR-STP and LTP when compared between the 10× and the 30× TBS experiments (figure 3*i,j*, orange and white symbols). In both cases, the GluN2D antagonist inhibited most of the NMDAR-STP (figure 3*k*) without affecting LTP (figure 3*l*). Thus, NMDAR-STP (14.8 ± 4.3%) that was induced with 10× TBS declined with a *τ* value of 1.3 ± 0.4 min to an LTP level of 19.2 ± 3.3% (figure 3*i*, orange circles). A similarly small (10.9 ± 4.0%) and fast-decaying (2.5 ± 1.5 min) NMDAR-STP was observed in the 30× TBS experiments with UBP145 in which a large LTP (29.6 ± 8.6%) was still being observed (figure 3*j*, orange squares). Such results suggest that both 10× and 30× TBS-induced NMDAR-STP are particularly sensitive to UBP145, resulting in a near-complete inhibition of NMDAR-STP without affecting LTP.

**Figure 4.**
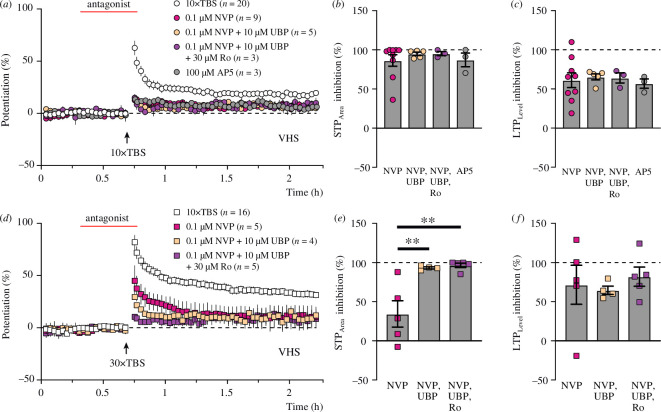
Incremental sensitivity of 30× TBS-induced NMDAR-STP to NMDAR antagonists. (*a*) Time courses of potentiation induced by 10× TBS in control experiments (white circles), in the presence of GluN2A-preferring antagonist NVP (pink circles), with NVP and GluN2D-preferring antagonist UBP145 (peach circles) and with NVP, UBP145 and GluN2B-preferring antagonist Ro (lilac circles). Grey circles show experiments with AP5. (*b*) NMDAR-STP_Area_ was inhibited to a similar extent when compared between the following four conditions (*p* = 0.75, ANOVA): 86.5 ± 7.3% for NVP alone, 95.0 ± 2.1% with the addition of UBP145 or 95.3 ± 2.6% with the addition of both UBP145 and Ro and 87.2 ± 8.8% for AP5. (*c*) LTP_Level_ was inhibited to a similar extent (*p* = 0.95, ANOVA) for the following four in-between group comparisons: 61.2 ± 9.4% (NVP), 65.5 ± 3.9% (NVP and UBP145), 64.1 ± 6.5% (NVP, UBP145 and Ro) and 56.9 ± 5.9% (AP5). (*d*) Time courses of potentiation induced by 30× TBS in the control experiments (white squares), in the presence of NVP (pink squares), with NVP and UBP145 (peach squares) and with NVP, UBP145 and Ro (lilac squares). (*e*) NMDAR-STP_Area_ was inhibited to a greater extent by a combination of either NVP and UBP145 (93.2 ± 1.3% inhibition, *p* = 0.0056, Dunnett’s multiple comparisons test) or NVP, UBP145 and Ro together (96.5 ± 2.8%, *p* = 0.0026, Dunnett’s multiple comparisons test) compared to only NVP (34.4 ± 16.9% inhibition, *p* = 0.0023, ANOVA). (*f*) There was no difference (*p* = 0.79, ANOVA) in the amount of inhibition of LTP_Level_ by NVP (71.6 ± 23.9%) compared to NVP plus UBP145 (64.6 ± 5.4%) or NVP, UBP145 and Ro all combined (82.0 ± 12.2%).

We were interested in whether combined application of the different antagonists would permit inhibition of the residual phases of plasticity, which were observed in figure 3. The residual LTP induced by the 10× TBS paradigm was unaffected by combining 0.1 μM NVP, 10 μM UBP145 and 30 μM Ro (6.7 ± 1.2%, lilac circles, figure 4), indicating that the residual LTP phase (~7%) is not dependent on NMDAR activation (figure 4*b,c*). We also tested 100 μM D-AP5 and found that it inhibited LTP to a similar extent (residual LTP = 8.1 ± 1.1%, *n* = 3, grey circles; figure 4*a,c*).

In contrast to the 10× TBS experiments, different combinations of the NMDAR antagonists produced a graded reduction in the slow-decaying NMDAR-STP in 30× TBS experiments (figure 4*d*), without further inhibition of the residual LTP (6–11%). Thus, the slowly decaying NMDAR-STP (31.2 ± 8.7%, *τ* = 17.8 ± 4.1 min), which was observed in the 0.1 μM NVP experiments (figure 4*d*, pink squares), was sensitive to additional application of 10 μM UBP145 (NMDAR-STP = 18.4 ± 1.7%, *τ* = 3.0 ± 0.7 min, peach squares) and could be even further inhibited by a combination of the three antagonists together (NMDAR-STP = 6.5 ± 2.9%, *τ* = 2.2 ± 1.2 min, lilac squares), suggesting that 30× TBS-induced NMDAR-STP involves the activation of more than one NMDAR subtype.

**Figure 5.**
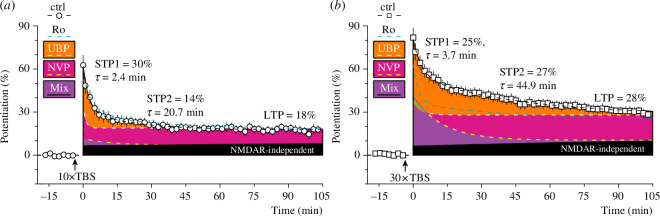
NMDAR-dependent potentiation components in the VHS. (*a*) Mean 10× TBS-induced control potentiation (Ctrl, white circles, *n* = 20) was fitted better with a double-exponential function (thick black dashed line, 90 min fit duration) than with single-exponential function (*F*-test, *F*_2,915_ = 5.739; *p* = 0.0033), producing estimates of fast- and slow-decaying NMDAR-STP (STP1 and STP2, respectively) and LTP. The fitted constants of the control potentiation are shown on the panel. 1 µM Ro did not inhibit NMDAR-STP or LTP induced by 10× TBS, and these results were also fitted with a double-exponential function (blue dashed line, mean data figure 3*e*). On the contrary, mean time courses of potentiation that were induced in the presence of 10 μM UBP145 (NMDAR-STP = 14.1%, *τ =* 1.1 min, LTP = 19%, white dashed line, mean data figure 3*i*) or 0.1 μM NVP (NMDAR-STP = 4.6%, *τ* = 12.5 min, LTP = 7%, yellow dashed line, mean data figure 3*a*) were better fit by single-exponential curves than by double, and both antagonists inhibited NMDAR-dependent potentiation. Amounts of potentiation that were inhibited by the antagonists are visualized with solid colours (areas above the fitted curves). UBP145 partially inhibited NMDAR-STP but not LTP (solid orange, area above white dashed line). In addition to the orange area inhibited by UBP145, 0.1 μM NVP inhibited a large amount of NMDAR-STP and fully inhibited NMDAR-dependent LTP (solid pink, area above yellow dashed line). The small, residual, NVP-insensitive component of STP (solid lilac) was inhibited by a mixture (mix) of UBP145 (10 μM), NVP (0.1 μM) and Ro (30 μM). Black area, derived by fitting single-exponential function to the mix of the antagonists (mean data figure 4*a*), visualizes NMDAR-independent component of LTP. (*b*) 30× TBS experiments are presented in the same way as 10× TBS experiments above. Once again, both the controls (white squares, *n* = 16, thick black dashed line, *F*_2,731_ = 7.938; *p* = 0.0004) and the experiments using 1 μM Ro (blue dashed line, *F*_2,225_ = 5.015; *p* = 0.0074, mean data figure 3*f*) were better fit with double-exponential functions, while 10 μM UBP145 (NMDAR-STP = 9.2%, *τ =* 3.3 min, LTP = 28%, white dashed line, mean data figure 3*j*) and 0.1 μM NVP (NMDAR-STP = 30.3%, *τ* = 12.8 min, LTP = 11%, yellow dashed line, mean data figure 3*b*) were better approximated by single-exponential functions. The orange component of NMDAR-STP above the blue 1 μM Ro line can be inhibited by both Ro and UBP145. The green solid inclusion area indicates the small amount of NMDAR-STP that is inhibited by UBP145 and preserved by NVP. The large, residual NVP-insensitive component of STP (solid lilac) is inhibited by the Mix of UBP145 (10 μM), NVP (0.1 μM) and Ro (30 μM). Black area, derived by fitting single-exponential function to the mix of the antagonists (mean data figure 4*b*), visualizes NMDAR-independent component of LTP.

Pharmacological segregation of the distinct potentiation components in the VHS, which were sensitive to NVP, Ro and UBP145, is shown in figure 5 to collectively illustrate the relationships between intensity of TBS, slow- and fast-decaying STP, LTP and the relative contribution of NMDAR subtypes. Notably, control NMDAR-STPs that were induced by 10× and 30× TBS were best approximated by double-exponential functions, while STPs that were recorded in the presence of either 0.1 μM NVP or 10 μM UBP145 were fitted best by single-exponentials, supporting the suggestion that NMDAR-dependent potentiation in VHS is a compound phenomenon that is composed of discrete phases of potentiation induced through graded activation of GluN2A, GluN2B and GluN2D-containing NMDARs.

## 4. Discussion

### (a) NMDAR-STP and LTP in ventral and dorsal hippocampus

NMDAR-STP and LTP are two types of NMDAR-dependent synaptic plasticity that are co-induced in the hippocampus by extracellular high-frequency stimulation of the Schaffer collaterals. Consistent with previous publications, we report here that both NMDAR-STP and LTP are smaller in the VHS than in the DHS [34,53–57]. We also confirm that higher levels of LTP can be achieved in VHS by increasing the number of theta bursts during the induction [73], although under our experimental conditions, LTP in VHS saturated at a lower level than in DHS. The lower levels of potentiation induced in the ventral hippocampus when compared to the dorsal could potentially be due to different levels of NMDAR expression. Notably, some studies are finding a decrease in the receptor levels [74,75], while others are reporting an increase in GluN1 and GluN2B expression in VHS [54]. On the other hand, pre-synaptic factors may also be responsible for the induction of lower levels of NMDAR-STP and LTP in the ventral hippocampus than in the dorsal, in that the mechanisms of their induction depend on basal probability of neurotransmitter release (*P*_*R*_). In support of such interpretation, PPF is reduced in ventral hippocampus when compared to dorsal [54,56,58–60,76–79], suggesting a high *P*_*R*_ under baseline conditions in VHS. The amplitudes of both NMDAR-STP and LTP are directly related to baseline levels of PPF with large initial PPF leading to large NMDAR-STP and/or LTP [23,80], while the decay time constant of NMDAR-STP is independent of the basal PPF [23].

In contrast to LTP [53–57], NMDAR-STP has not been previously characterized in VHS in detail, and here we show that similar to the dorsal hippocampal NMDAR-STP [23,24,33,35], the decay of NMDAR-STP in VHS requires pre-synaptic activity. Hence, when stimulation is stopped for 30 min after the induction, NMDAR-STP remains stored in VHS and its decay resumes only when the stimulation is re-commenced, indicating that the process of decay is not dependent on the overall magnitude of NMDAR-STP expression. The results also demonstrate that increasing the number of TBS in an induction protocol increases the duration of NMDAR-STP. This observation is in line with the previous study in DHS, showing that it is the number of stimuli in a tetanus that regulates the duration of NMDAR-STP [23]. Thus, in VHS, 10× TBS induces a fast-decaying NMDAR-STP and 30× TBS induces a slower-decaying NMDAR-STP, supporting previous observations that two kinetically [27] and pharmacologically [35] different forms of NMDAR-STP can be induced within the hippocampus and in other limbic structures.

### (b) NMDAR dependence of ventral NMDAR-STP and LTP in 10× TBS experiments

Fast- and slow-decaying NMDAR-STP (termed STP1 and STP2, respectively) and LTP rely on activation of different NMDAR subtypes in DHS, and by using the same NMDAR subunit-preferring antagonists as characterized previously (figure 1*g,j* and table 1 [35]), we have investigated here whether the induction of NMDAR-STP and LTP in VHS could also be dissected pharmacologically while using the 10× TBS paradigm. 10× TBS induces saturated NMDAR-STP and LTP in DHS (figure 1*c*; see also Refs. [45,62]) and submaximal NMDAR-STP and LTP in VHS (figure 2*i*). We expected fast-decaying NMDAR-STP and LTP to be sensitive to NVP and slow-decaying NMDAR-STP to be sensitive to Ro and UBP145.

In accordance with our predictions, we found that the fast-decaying NMDAR-STP and LTP induced by 10× TBS were particularly sensitive to the GluN2A antagonist NVP (100 nM). NVP inhibited both types of potentiation close to their maximal extent, leaving a small NMDAR-independent LTP (figures 2*a,* 3 and 4*a*). The fast NMDAR-STP was also inhibited by the GluN2D antagonist UBP145 (10 μM), which did not affect the induction of LTP (figures 2 and 3). Such inhibition of the fast component of NMDAR-STP by UBP145 was not observed in DHS, where UBP145 preferentially inhibited the slow component of NMDAR-STP (figure 1*g* [35]). Thus, inhibition of the fast component of NMDAR-STP by UBP145 may be specific for the ventral hippocampus. In the VHS, the effects of NVP and UBP145 on the fast NMDAR-STP might be due to their inhibition of di-heteromeric GluN2D-containing receptors, or tri-heteromeric receptors containing both GluN2A and GluN2D subunits, in addition to the obligatory GluN1. The differential effect of NVP and UBP145 on LTP in VHS excludes involvement of GluN2Ds in LTP induction, just like in the DHS [35].

Notably, a GluN2B-selective concentration of Ro 25-6981 (1 μM) had neither an effect on NMDAR-STP nor on LTP, excluding the involvement of this subunit in the 10× TBS experiments (figures 2*e* and 3*a*). This appears in stark contrast to the published experiments in DHS, where 1 μM Ro decreases the decay time of NMDAR-STP without affecting the induction of LTP [35]. However, 10× TBS in the DHS induces both fast and slow components of NMDAR-STP, and 1 μM Ro inhibits only the slow component (figure 1*g*). Such slow component of NMDAR-STP is less pronounced in 10× TBS experiments in VHS (figures 2*e* and 3*a*). Increasing the concentration of Ro to 30 μM inhibited the induction of both NMDAR-STP and LTP, producing very similar effects as 100 nM NVP on its own (figures 3*a,e* and 4). Ro does not inhibit GluN2D receptors but can inhibit GluN2A at high concentrations [35,66]. We have previously noted that at concentrations above 10 μM, Ro starts inhibiting some GluN2A-containing di-heteromeric receptors, with about 25% inhibition at 30 μM [35]. It therefore seems possible that the effects of high concentrations of Ro on NMDAR-STP and LTP are due to inhibition of NMDARs that contain GluN2A subunits, either in di- or in tri-heteromeric combinations. To the best of our knowledge, Ro has not been tested on tri-heteromeric receptors (please see [81,82] for information on related compounds, such as ifenprodil and CP-101-606).

In summary, considering the cumulative results of the 10× TBS experiments with the three antagonists, the inhibition of the fast NMDAR-STP in 10× TBS experiments might be mediated by inhibition of GluN2A/2D-containing NMDARs (sensitive to NVP, UBP145 and high concentrations of Ro), while the inhibition of LTP could be mediated by inhibition of GluN2A- or GluN2A/2B-containing receptors (sensitive to NVP and high concentrations of Ro).

### (c) NMDAR dependence of ventral NMDAR-STP and LTP in 30× TBS experiments

The slow, 30× TBS-induced NMDAR-STP appeared to be pharmacologically distinct from the fast 10× TBS-induced NMDAR-STP. Thus, 100 nM NVP did not inhibit NMDAR-STP completely but preserved a large slowly decaying component of NMDAR-STP (figures 2*b* and 3). LTP, however, was inhibited by NVP to roughly the same extent as in the 10× TBS experiments (figures 2*d* and 3*b*), which means the NMDAR subunit that mediates induction of the slow NMDAR-STP does not contribute to the induction of LTP.

UBP145 inhibited the slow, 30× TBS-induced NMDAR-STP completely, suggesting that GluN2D receptors are involved in its induction in VHS (figures 2*j–k* and 3*b*). These results show that while UBP145 inhibits both the fast- and the slow-decaying NMDAR-STP without affecting LTP (figures 2*l* and 3*b*), NVP inhibits only the fast component of NMDAR-STP, as well as LTP. Such results suggest the involvement of an additional subunit in the 30× TBS induction of NMDAR-STP when compared to 10× TBS, which could be GluN2B, as it has the lowest affinity to NVP when compared to the other subunits. In support of that conclusion, 1 μM Ro decreased the decay time constant of NMDAR-STP in 30× TBS experiments and did not affect the induction of LTP (figures 2*f–h* and 3*b*); this effect is similar to the effect of 1 μM Ro on NMDAR-STP in DHS (figure 1*g*), as discussed above.

Increasing the concentration of Ro to 30 μM in the 30× TBS experiments inhibited both NMDAR-STP and LTP, similar to the results in 10× TBS experiments in VHS, and also in the DHS, as published previously [35]. The amount of LTP that was inhibited by 30 μM Ro was significantly larger in the 30× TBS than in 10× TBS experiments. However, this increased sensitivity of LTP to 30 μM Ro is unlikely to indicate the involvement of di-heteromeric GluN2B-containing receptors in the induction of LTP, as it remained insensitive to 1 μM Ro (figure 5*b*). We therefore currently believe that a pharmacologically homogeneous population of NMDARs, composed of either di-heteromeric GluN2A-containing receptors or tri-heteromeric receptors that contain both GluN2A and GluN2B subunits, mediates induction of LTP in both 10× TBS and 30× TBS experiments. On the other hand, considering the effects of Ro, we have to note that this antagonist has a complex allosteric mechanism of action: it does not produce 100% inhibition of di-heteromeric GluN2B-containing NMDARs [35], and it can even facilitate GluN2B responses at low agonist concentrations [35,66]. Therefore, the effects of Ro on synaptic plasticity may be difficult to interpret, involving changes in efficacy and contribution of spare receptors.

### (d) Segregation of the fast and slow NMDAR-STP and LTP in VHS

Although, as discussed above, we cannot be completely certain about the exact composition of the LTP-inducing NMDARs (i.e. GluN2As versus GluN2A/Bs) in VHS, we can still be confident that these receptors are pharmacologically different from the receptors that mediate the induction of NMDAR-STP. The fast NMDAR-STP in VHS is likely induced by NMDARs that contain GluN2A and GluN2D subunits, while the slow NMDAR-STP is induced by NMDARs containing GluN2Bs and GluN2Ds. Importantly, the two types of NMDAR-STP in VHS differ not only from each other in terms of NMDAR subunits involved but also from LTP, which does not require the involvement of GluN2Ds (figure 5). Such complete segregation of the fast STP1, the slow STP2 and LTP was not that obvious in DHS (figure 1*g–j* [35]) where STP1 lacked sensitivity to inhibition of GluN2Ds and was more akin to LTP in terms of sensitivity to GluN2As. Sensitivity of the slow STP2 to inhibition of GluN2B/2D subunits is shared between the dorsal and the ventral hippocampus. The effects of NMDAR inhibitors in the VHS are in line with the previously published results using GluN2 subunit potentiators (figure 1*e,f* [34]). Here, the GluN2A/2B-preferring PAM UBP714 potentiated induction of LTP and decreased the decay time constant of NMDAR-STP, while the GluN2C/D-preferring PAM CIQ increased the amplitude of NMDAR-STP and slowed its decay, without affecting induction of LTP. Activation of the slow NMDAR-STP requires prolonged tetanization in the VHS, which might suggest that higher concentrations of glutamate (or glutamate spillover) are required to activate NMDARs that are responsible for its induction. Such NMDARs might be located either extra- or peri-synaptically, on either pre- or post-synaptic terminals, and future investigations will have to be conducted in order to determine sub-cellular locations of these receptor complexes.

### (e) Final remarks: wider pharmacological implications for the study of synaptic plasticity

The results presented in this publication show that the sensitivity of both NMDAR-STP and LTP to NMDAR antagonists (NVP and Ro) changes dependent on the duration of TBS and the magnitude of synaptic plasticity induced. Such differential sensitivity, which corresponds to the level of agonist-evoked biological effect, is likely to complicate comparisons between different studies that use single concentrations of antagonists to investigate synaptic plasticity in preparations without clearly defined maximal effects. Many previous studies used NVP and Ro to infer conclusions about NMDAR subunit involvement (or lack of involvement) in regulating the induction of LTP and LTD, and we have discussed the disparity of the results in earlier publications [5,34,35]. On this occasion, we can only stress that our current observations extend beyond the use of NMDAR antagonists and that without comparing ‘like with like’ we shall probably be discussing the basic principles of pharmacology during the 60th celebration of LTP.

## Data Availability

All data and their analyses are included in this article. Any additional information is available from the corresponding author on reasonable request.
